# WikiArtVectors: Style and Color Representations of Artworks for Cultural Analysis via Information Theoretic Measures

**DOI:** 10.3390/e24091175

**Published:** 2022-08-23

**Authors:** Bhargav Srinivasa Desikan, Hajime Shimao, Helena Miton

**Affiliations:** 1 Computer and Communication Sciences, École Polytechnique Fédérale de Lausanne (EPFL), 1015 Lausanne, Switzerland; 2 Department of Economics, McGill University, Montreal, QC H3A 0G4, Canada; 3 Santa Fe Institute, Santa Fe, NM 87501, USA

**Keywords:** cultural analysis, dataset, information theory, deep learning, color representations, style extraction, analysis framework, art history

## Abstract

With the increase in massive digitized datasets of cultural artefacts, social and cultural scientists have an unprecedented opportunity for the discovery and expansion of cultural theory. The WikiArt dataset is one such example, with over 250,000 high quality images of historically significant artworks by over 3000 artists, ranging from the 15th century to the present day; it is a rich source for the potential mining of patterns and differences among artists, genres, and styles. However, such datasets are often difficult to analyse and use for answering complex questions of cultural evolution and divergence because of their raw formats as image files, which are represented as multi-dimensional tensors/matrices. Recent developments in machine learning, multi-modal data analysis and image processing, however, open the door for us to create representations of images that extract important, domain-specific features from images. Art historians have long emphasised the importance of art style, and the colors used in art, as ways to characterise and retrieve art across genre, style, and artist. In this paper, we release a massive vector-based dataset of paintings (WikiArtVectors), with style representations and color distributions, which provides cultural and social scientists with a framework and database to explore relationships across these two vital dimensions. We use state-of-the-art deep learning and human perceptual color distributions to extract the representations for each painting, and aggregate them across artist, style, and genre. These vector representations and distributions can then be used in tandem with information-theoretic and distance metrics to identify large-scale patterns across art style, genre, and artist. We demonstrate the consistency of these vectors, and provide early explorations, while detailing future work and directions. All of our data and code is publicly available on GitHub.

## 1. Introduction

The curation and dissemination of large cultural datasets have presented cultural theorists and analysts with an unprecedented opportunity for large-scale digital historical excavation. Image-based datasets have, in particular, grown over the last decade, along with an increase in sophisticated computing machinery to analyse these datasets. While most such datasets are built for the purpose of training computer-vision models for tasks such as image classification, object recognition, and OCR, a handful of datasets have been curated for the purpose of analysis of the dataset itself, as opposed to training other models. One such popular dataset is *WikiArt*, which contains nearly 250,000 artworks by 3000 artists, localized in eight languages, featuring artwork from over a 100 countries. While there exist other datasets that curate artworks for analysis, for example, the National Gallery of Art dataset, WikiArt ([1,2]) serves as an especially valuable resource for its extensive coverage of artworks over a millenium, annotated by artist, date, genre, and style. *WikiArt*’s wide adoption in the computer-vision community, as well as its use in multi-modal datasets, such as *Wiki Art Emotions* [3], make it a valuable dataset for cultural analysis.

However, most existing literature in the computer-vision community uses *WikiArt* for the purposes of image classification or synthesis, as opposed to cultural or historical analysis. Further, existing work on aesthetics and visual content in art has largely used object-detection and similar approaches, but art historians bemoan that most computer-vision applications consider *“relatively unimportant and unproblematic issues, if not given.“* [4]. Art historians are not looking for vision-based systems to automate discovery or analysis, but, rather, for systems to aid in search, retrieval, and organisation [5].

In this paper, we intend to bridge this gap by releasing a dataset of aggregate vectors and representations to perform such organisation, as well as aid in large-scale pattern recognition that may further aid art historians and cultural analysts with their research, and truly take advantage of the powers that modern computation provides. While notions of entropy and complexity have been used previously to study art history ([6,7]), they have been singular studies to showcase a specific method, as opposed to a set of tools presented to art historians and cultural theorists. Our dataset and aggregate vector representations both showcase the early steps and possibilities of our framework, while also allowing for powerful organisational capabilities to aid other scholars.

Our vector representations of paintings are powered by modern deep-learning approaches, which is the current state of the art for image-based applications. These neural network models are trained to extract *style* representations, allowing for paintings to be indexed by their artistic style. Moreover, these vectors may be aggregated for artists, genres, styles, and time periods, allowing for analysis at different levels.

While deep-learning methods excel in identifying patterns of pixels and their texture, they fail in using color information when representing images [8]. Color, however, remains a key aspect of fine art and painting. For example, it is used to characterise certain artist’s stylistic periods, e.g., Picasso’s blue phase, and certain art movements used particularly vivid colors, such as Fauvism. To overcome this failing of neural networks, we also create perceptually uniform color representations of paintings that capture the distribution of colors in an image. Similar to the style representations, these vectors can also be aggregated by artist, style, genre, and time period.

By releasing our *WikiArtVectors* dataset of style and color representations of paintings, and the corresponding aggregate representations for artists, styles, and genres, we fill a crucial gap in the art history, cultural analytics, and digital humanities literature. These representations can be used for indexing, search, retrieval, as well as information-theoretic inspired analyses. As the color representations take the form of binned probability distributions of color, and the style representations as latent distributions of style, these representations can be extended easily in the context of entropy-based approaches.

We start with a review of the existing literature on computation and art to situate our work, and then describe our vector dataset, some early explorations, and the potential for cultural and historical analysis.

## 2. Computation and Art

Since the 2010s, there has been an explosion in the number of papers exploring art forms using computational methods ([5,6,7,9,10,11,12,13,14,15,16,17,18,19,20,21,22,23,24,25,26,27,28,29,30,31,32,33,34,35]). This has been due to the more formal organisation of image-based datasets, and the large-scale use of neural-network- and data-science-based approaches for image analysis. In this section, we detail existing work in the computational analysis of art, and also focus on analysis using deep learning, color, and information theory.

### 2.1. Computational Analysis of Art and Aesthetics

Large scale analyses of artworks using computational techniques have explored many aspects of art, such as quantifying creativity in art networks [11], or creativity in the content of fine art [12]. Computational analysis is not limited to large-scale images, but also includes different forms of art, such as clay-practice [13], where researchers have explored the dynamics of personal artistic style while tracking the artist’s use of carving knives.

Earlier computer analysis of art [14] used methods borrowed from bio-medical imaging that captured properties of color, and pixel intensity and distribution. Another domain of inquiry is *aesthetics*, where methods to detect lines, contours, and signs are identified to measure aesthetic content ([15,16]). Using both deep-learning and traditional approaches, identification of such symbols and signs, as well as actors, goes towards building semantic interpretations of paintings [17].

### 2.2. Deep Learning and Art/Paintings

Most modern approaches to the analysis of art and paintings use deep learning as the foundational approach. Deep-learning approaches to the analysis of art are often centered on classification tasks, such as artist prediction, genre prediction, style prediction, or period estimation ([21,22]). Such approaches are often integrated with multiple parallel predictive tasks for better performance ([19,20]).

Outside of prediction tasks, capturing or representing artistic style is a popular task involving deep learning and art, popularly referred to as *style transfer* ([18,24,25]). In such approaches, the training objective for the neural algorithm is to transform a target image (e.g., a landscape) in the artistic style of another image (e.g., a Van Gogh impressionist piece). During this process, the neural network weights learn a *style representation* of an image, which can be applied as a matrix transformation to the target image. Such representations can be used for multiple downstream tasks, such as image retrieval—indeed, we extensively use such approaches by creating our own *style vectors*.

We note that most deep-learning-powered approaches to artwork and paintings focus on predictive or indexing tasks.

### 2.3. Color Computation and Art

While machine-learning and deep-learning approaches often capture stylistic features, shapes, lines, objects and contours, they are not well-suited to capture color information, which remains a crucial aspect of the semantic and cultural organisation of art [8]. Most computational approaches to analysing color in artwork involve first- and second-order statistics of color distributions ([28,30,32]), demonstrating that artists differentiate themselves with their usage of color, or by drawing comparisons to real-world images’ color distributions.

Other approaches include using the color palette of paintings as the object of analysis ([29,31]), with work describing the gamut of colors used in fine art, as well as the psychological determination of relevant colors. Few approaches [33] use color-based vectors to perform classification and influence analysis.

While using color as a way to investigate art is a common approach in critical art studies and art history, this approach is not as developed in the context of computational approaches.

### 2.4. Machine Learning and Information Theory for Historical and Cultural Analysis

While most developments in the use of computational approaches to fine art and paintings have been geared towards classification and prediction, there exists a strain of work that attempts to contribute to art history and cultural analysis using such tools. The notion of using entropy, complexity, and information-theoretic approaches has been tried with varied approaches. Work by Sigaki et al. [6] used local spatial patterns to determine complexity, and to chart the dynamical behaviour of this measure over millennia. Silva et al. [7] used normalized compression values to operationalise complexity, and sought to identify patterns and fingerprints of artists and genres across 4266 paintings.

Another approach to such analysis is using deep-learning-based approaches to attempt to track similar patterns and trajectories of style over centuries of art. Ahmed et al. [9] trained a convolutional neural network to classify images and analyse the learned representations of the neural network weights to reveal the primary dimensions neural models focus on. Another approach is to use generative adversarial networks [10], which are neural networks trained to generate images that mimic art styles or genres. Such models can be used in tandem with temporal data to predict future art movements and styles [23].

While such approaches often fall within the domains of complexity or computer science, art historians have different expectations and hopes for computer-vision applications for art history [34]. While images and objects in art can shed light on the evolution of art, the current emphasis within the discipline is on art history’s *discourse* rather than objects [5]. An effective interdisciplinary approach would aim to lessen the gap between the current state of the art in computational approaches to art history and historians’ expectations.

## 3. *WikiArtVectors*: Vectorisation and Feature Extraction

We aim to bridge the gap in computational approaches to art history and cultural analysis by releasing individual vectors and aggregate vectors for the paintings in the WikiArt dataset (We release our dataset and demo notebooks on GitHub: https://github.com/bhargavvader/WikiArtVectors, accessed on 9 June 2022). This dataset can serve multiple purposes for different communities pursuing multi-disciplinary approaches. In this section, we detail our process for creating and aggregating these vectors.

### 3.1. CNN Based Style Vectors

To extract stylistic information from paintings, we use the suite of methods detailed in the *style transfer* literature ([18,24,25]). Specifically, we use PyTorch [36] to extract the style representation from a VGG19 convolutional neural network [37]. This is achieved by extracting the *gram matrix*, which is a matrix created by multiplying a given matrix by its transposed matrix. In our case, the *gram matrix* is created from the feature maps of the pre-penultimate layer of the convolutional neural network when given the artwork as input. This allows us to extract a unique representation for each artwork. (We note here that we choose the pre-penultimate layer, as suggested by the style-based retrieval results in [24]. Our code allows functionality by extracting style information from any layer of the CNN).

The extracted gram matrix, however, is of 4096 dimensions, which is quite large for rapid analysis and is expensive to store. We follow the lead of the work reported in [24], where the authors tested various PCA dimension reductions of the extracted gram matrix for their performance in style-based image retrieval. For our purposes, we use incremental PCA to reduce our gram matrix to 256 dimensions. This reduction allows us to retain the discriminatory power of the style representations while allowing them to be readily used for analysis and retrieval.

### 3.2. Human Perceptual Color Distributions

To complement our neural-network-based style representations, we extract human perceptual color information from the artworks. We again note that neural networks do not specifically extract color-based information from images, focusing instead on patterns of pixels and texture [8]. To ensure that our representations capture multiple aspects of artwork, we measure color information using a state-of-the-art transformation of colorspace that accurately captures human color perception (referred to as a *perceptually uniform colorspace*) [26,35]. We use the *comp-syn* package to extract these color representations [27], where each artwork is represented by an eight-dimensional *color-distributional* vector. This representation has been shown not only to extract key color properties in images, but also to capture multi-modal information on the linguistic and cultural aspects of color ([26,27]).

### 3.3. Vector Aggregation and Organisation

After extracting every image through our style and color extraction pipelines, we release a dataset mapping every artwork (based on an ID and the title of the artwork) to its style vector, color vector, artist, genre (as designated by WikiArt), style (as designated by WikiArt), and year of the artwork. We discard any artworks without all of this information.

This mapping presents many opportunities for aggregation. Our first level of organisation is to aggregate vectors by artist and the year associated with the artwork. For example, it may be, e.g., Van Gogh, 1888, where this entity is then linked to an aggregate style vector and color vector, created by averaging all the individual artworks associated with the artist in that year. We similarly create aggregate vectors of style (e.g., Landscape, 1880), and genre (e.g., Impressionism, 1890). We also aggregate vectors by decade, as well as all the artworks within a category.

We note that there are multiple options for aggregating the style and color vectors, and that these options have qualitative and quantitative consequences for the resultant vectors. Our default option aggregates artworks on our three main categories of artist, genre and style, by calculating the average vector at each index, based on the temporal length of choice. It is also possible to manually aggregate vectors of selected artworks by index. We also include options for weighted averaging of artworks, as not all artworks may necessarily be deemed equally important in characterising the artist or genre.

We include such flexibility and precision in our aggregation process as the method of aggregation would greatly influence the resultant vectors. For example, treating all artworks with equal weighting may result in obfuscating certain properties we wish to account for. We do not prescribe a ‘correct’ way of aggregating vectors as it depends heavily on the downstream task at hand or the nature of the art retrieval task at hand. To offer comprehensive options for smooth averaging techniques, we construct our API to allow for various smoothing options (e.g., Gaussian smoothing).

These multiple levels of aggregation allow for exploration and similarity metrics at different levels of granularity. The inclusion of individual vectors for every artwork also allows for more fine-grained analysis and retrieval. Such organisation allows for maximum utility for both art historians and those interested in computational cultural analysis.

## 4. Entropy and Information Theoretic Measures

The advantage of such multi-dimensional representations is that they can be operationalised and viewed as probability distributions, allowing us to use entropy-based information-theoretic measures. Previous work [38] has demonstrated how the variance in the structure of embedding representations can be captured using cosine similarity, allowing us to use a framework of entropy on our representations.

In the following sections, when we discuss the distance between two artwork/aggregate color representations, we refer to the Kullback–Leibler divergence between the two representations. As each color representation is a binned eight-dimensional probability distribution [27], measures such as the KL divergence or the Jensen–Shannon divergence can measure the statistical distance between two artwork color distributions.

If the first artwork representation or aggregate representation is *P*, and the second is *Q*, the distance is defined as:

(1) $$KL\left(P||Q\right)=\sum_{x\in X}P\left(x\right)\log\frac{P(x)}{Q(x)}$$

When we discuss the distances between two artwork/aggregate style representations, we refer to the cosine distance between the two representations. If the embedding representations are represented as vectors **t, e**, this distance is:

(2) $$\cos\left(t,e\right)=\frac{te}{∥t∥∥e∥}=\frac{\sum_{i=1}^{n}t_{i}e_{i}}{\sqrt{\sum_{i=1}^{n}{\left(t_{i}\right)}^{2}}\sqrt{\sum_{i=1}^{n}{\left(e_{i}\right)}^{2}}}$$

where *n* is the total number of dimensions of the representation.

## 5. Vector Exploration and Validation

While the full WikiArts dataset contains over 250,000 artworks, only a subset of this dataset has full meta-data that allows us to create aggregate representations. On filtering for artworks that have information on the title, year of artwork, artist, genre, and style, we are left with less than half the original dataset.

Our dataset contains a total of 68,094 artworks and their corresponding vectors, with 1626 unique artists, 132 unique styles, and 42 unique genres (We release our dataset and demo notebooks on GitHub: https://github.com/bhargavvader/WikiArtVectors, accessed on 9 June 2022). There are a total of 24,857 temporal artist vectors, 6055 temporal style vectors, and 6402 temporal genre vectors at different levels of aggregation. In this section, we will showcase some of the use-cases of such a framework. In our demo Jupyter notebooks, we showcase some of the use-cases of our vectors and representations. We broadly demonstrate three use-cases: (1) retrieving similar artworks, (2) clustering similar entities, and (3) measuring change over time. We note that these use-cases only touch the surface of all the possibilities with this vectorised data-set.

We also note that such representations must be validated and grounded by art historians and cultural theorists themselves—while the representations themselves are validated in the original research articles describing the techniques, they are validated on tasks (e.g., artist prediction, human color perception) that may not be relevant to the art historian. Frameworks of validation for such artwork representations do not currently exist and would represent a promising future research direction.

### 5.1. Retrieving Similar Artworks

**Figure 1:**
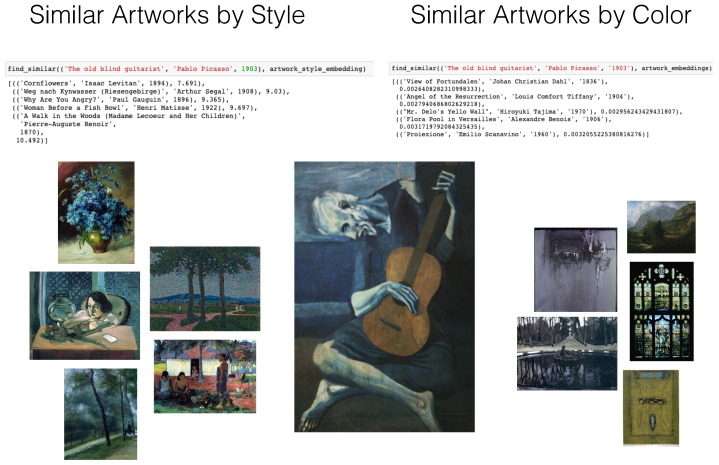
Retrieving similar artworks by style and color representations.

In our first demo notebook, we demonstrate a few functions that work with our vector dataset to perform image-retrieval and organisation tasks. Our dataset can search across artist, genre, style, and time period, retrieving similar images in each of these categories. In Figure 1, we show search results for Picasso’s *The Old Guitarist*, painted during his *blue period*, based on color similarity, and style similarity. We note that what is retrieved is the artwork meta-data, and not the image of the artwork itself.

In the example, we see how style and color representations return different artworks, with similar color artworks featuring shades of blue and yellow, and similar style artists retrieving largely post-impressionist paintings.

These searches can also be temporal, and we can adjust the search to return any number of paintings ranked by proximity to the search criteria. If searching by style, we use the cosine distance to measure how close two artworks are in the style space. The cosine distance is a popular metric when working in such multi-dimensional spaces, though our framework is modular and can use any distance metric. When searching by color, we use the Jensen–Shannon divergence measure, an information-theoretic measure, and our representations can be thought of as probability distributions of color. Similarly, the use of measure here is flexible, and any information-theoretic measure, such as KL divergence, would work reasonably well. We also note that is possible to search across both color and style by appending the vectors, or by searching for intersections in artworks across both the styles, an option we have allowed through our code.

This use-case is intended to aid art historians with search and labelling, and to explore the complex space of artworks and their relations in a dynamic way.

### 5.2. Clustering and Dimensionality Analysis of Entities

This representation-powered approach can also be useful in clustering entities in style space or color space. We allow for functionality to cluster entities by artist, style, and genre, and for temporal filters. Our demo notebooks showcase visual clustering, via methods such as tSNE and UMAP, which preserve local and global structure. Clustering can be performed at the individual artwork level or at the artist aggregate level. Such visualisations can help explore the *spread* of the artist’s work in the style or color spaces, which can be verified by information-theoretic measures.

**Figure 2:**
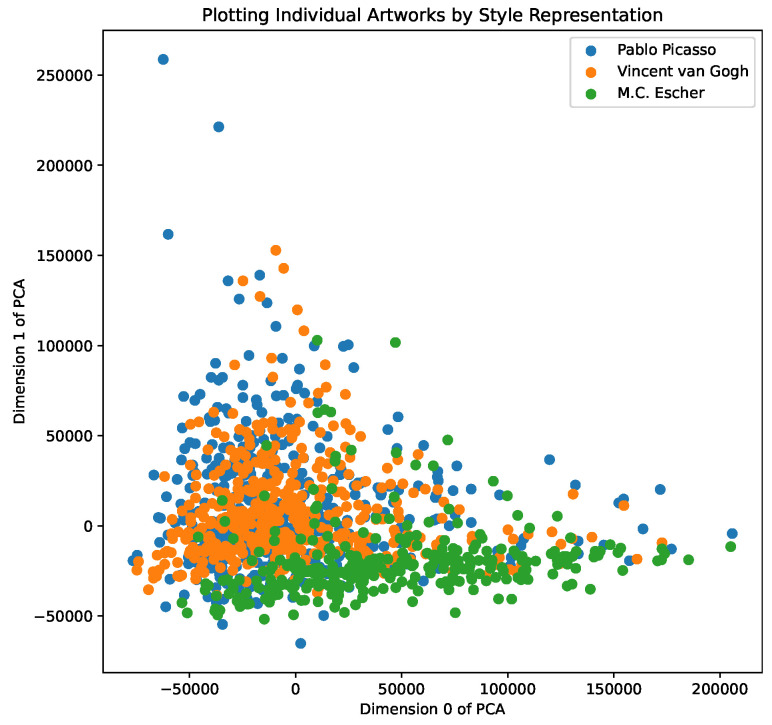
PCA of individual artworks’ style representations.

**Figure 3:**
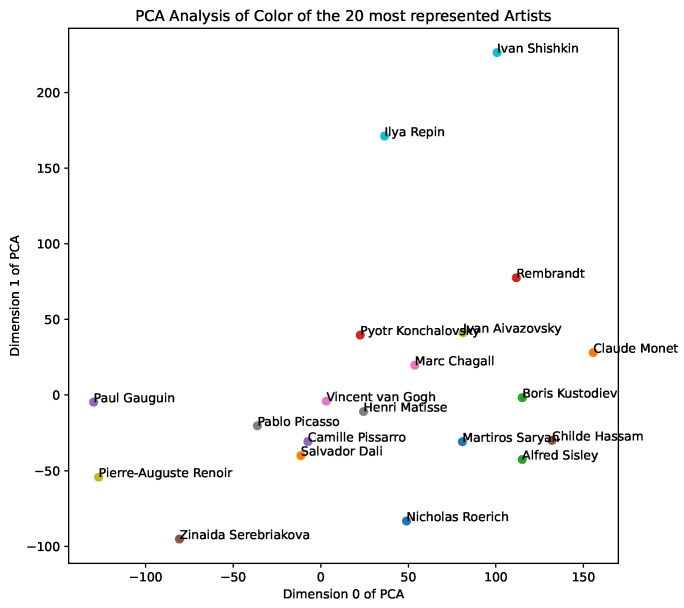
PCA of aggregate artists’ color representations.

We also include functionality for dimensionality analysis using PCA, extending the results in earlier work [9], and find that the first two dimensions of the PCA of style representations capture 60% of the variance, allowing us to potentially isolate markers of art style, as described in [9]. In Figure 2, we show a PCA dimensionality analysis across individual artworks by Picasso, van Gogh, and M.C Escher, allowing us to explore their overlap and spread in the *style space*, and, in Figure 3, we show a PCA analysis of the aggregate color representations of the 20 most represented artists—we note clusters of impressionist painters, as well as landscape painters. We include further results of PCA analysis and visualisations in our demo notebooks.

We note that, as we create aggregates with larger time periods, our representations become *flatter*, and it is more difficult to find meaningful patterns with large averaged vectors. In such scenarios, we recommend using custom vectors aggregated by smooth techniques. Our demo notebooks include multiple visualisation options and sample visualisations.

### 5.3. Measuring Entropy and Change over Time

Using divergence and distance measures, we can now use style and color representations to measure how artists, styles, and genres have changed their color and style across their artworks. We demonstrate in our demo notebook how we can use such measures to explore change in the make-up of artworks and their dimensions over time. Our category entropy measure samples 1000 pairs of artworks within an artist, category or style to measure the spread of artworks in the style and color spaces. For measuring change, we aggregate representations by time period and measure the cosine distance (for style space) or divergence measures (color space) between them. We provide sample code to perform such operations in our demo notebooks.

**Figure 4:**
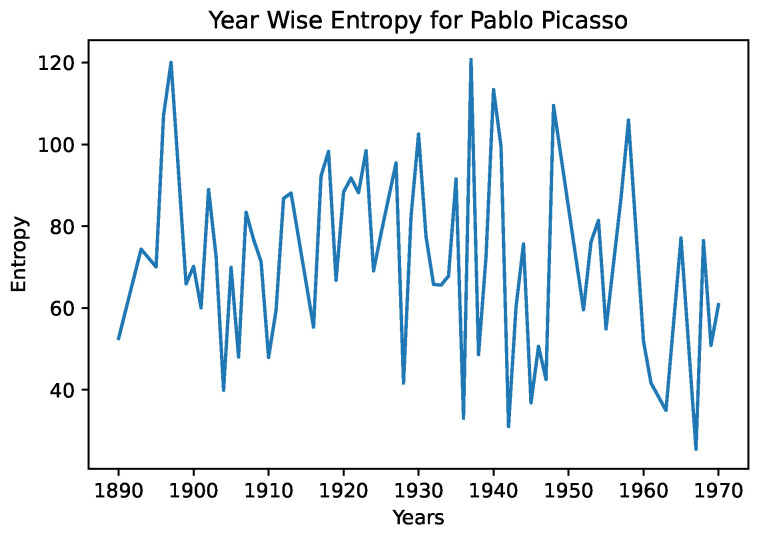
The distance between successive-year style representations for Pablo Picasso.

**Figure 5:**
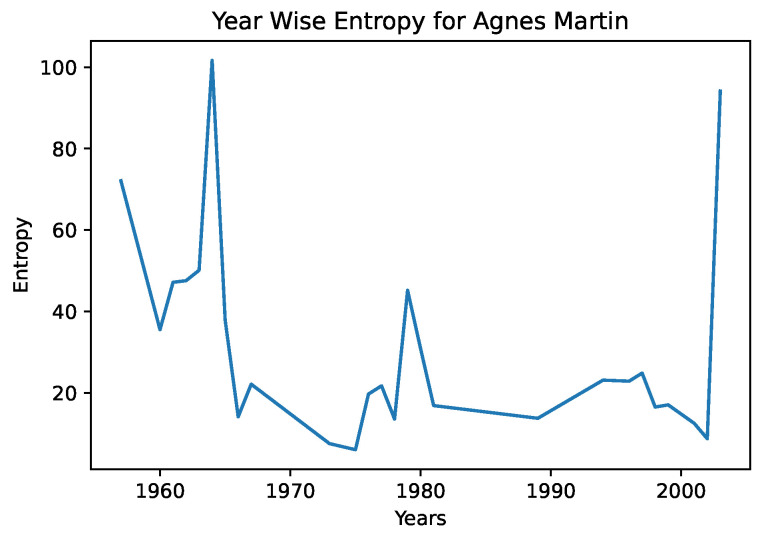
The distance between successive-year style representations for Agnes Martin.

In Figure 4 and Figure 5, we see the year-wise style entropy for Picasso, and Agnes Martin. In this case, artworks were aggregated by year, and the distance between successive years representations were calculated, to search for changes in average artstyle. We note in the entropy diagram for Picasso that the average distance is higher, suggesting that he frequently changed art styles. In the case of Agnes Martin, a celebrated Canadian-American abstract painter, we see sharp changes earlier and later in her artworks, and a lower distance from the 1970s–2000s. However, on closer inspection of Agnes Martin’s work, we note that she played extensively with color and arrangements, pioneering the field of abstract expressionism. This differs greatly from the object-focused artwork of Picasso—a CNN would be better able to capture the style and texture of cubism than abstract expressionism. This example serves to illustrate that the entropy measures must be interpreted in a grounded fashion and in conjunction with color representations.

## 6. An Information-Theoretic Framework for Cultural Analysis

Our dataset represents artworks as multi-dimensional vectors, and allows us to conduct historical and cultural analysis, as it provides quantitative descriptions of two key features of artworks: style and color. It is intended to be a resource for art historians, digital humanists and cultural evolutionists alike. It provides researchers from diverse fields with structured, high-quality information on a large sample of artworks. Information theory provides methods and measures that enable exploitation of this dataset, and other similar resources, to answer important questions about the evolution of art and culture at large.

### 6.1. What Can We Explore?

There are several possible uses of our dataset. The methods we introduced as use-cases, and those described further in this section, can be used to test and discuss dynamics that are known from art history, or broader hypotheses about the creativity and dynamics of innovation. Additionally, because our dataset provides probability distributions, it lends itself to being analyzed using information-theoretical measures (e.g., Kullback–Leibler divergence, Jensen–Shannon divergence, Granger causality, etc).

### 6.2. Complementarity with Other Work

There is substantial complementarity between our work and other work, whether it bears on culture at large, art history, or specifically computational studies of large-scale data.

With respect to other computational methods for the analysis of large-scale datasets, our dataset can be used to cross-validate other methods, such as work on aesthetic complexity [39]. Our dataset and methods can be employed for similar uses as outlined in [39], including being related to human judgments, evaluating individual artists’ trajectories over their careers, or being used for authorship and style attribution.

Another form of complementarity is with the categories and understanding of art history created by art historians themselves. Our dataset could be used to recreate and validate their categories and constructions, and vice versa, whenever art history categories do not match the ones obtained from computational analyses; this would be informative with respect to which dimensions computational methods are unable to capture.

Our dataset could also be combined with other datasets of artists’ networks or biographical information (such as their locations, e.g., [40]). This would allow the addition of geographic and social dimensions to the questions we have focused on.

### 6.3. Art History and Dynamics of Innovations and Influence

Innovations, their spread, and how one innovation or innovator influences others, are important questions in understanding what shapes cultural dynamics.

Previous work has used information-theoretic measures, as a means of measuring the influence from one event or cultural production to another. For instance, the Kullback–Leibler divergence (KLD) has been used to track innovations and influences at the level of word-use patterns during the French Revolution [41].

We anticipate that, from the dataset we introduced here, several types of influences could be similarly explored, quantified, and tested for, at the different levels of individual artworks and aggregates (artists, movements). This could relate to the influence of one painting on another, of one artist on another, but also of one specific artwork on others, for instance, concerning specific paintings or artists that have been suggested to be specifically influential for particular artistic movements. This should, obviously, be undertaken in concert with art historians, to make the best use of their knowledge, and increase the relevance of the approach to their own questions.

Another form of influence relates to voluntarily departing from previous artworks or movements, rather than adopting some elements of their style. Again, it should be possible to detect such dynamics using information-theoretic measures.

There is also growing evidence that change and innovation in the arts (at least for painting and literature) are driven by cohort, or generational, effects ([42,43]). Cohort effects can be diagnosed when the birth year of the artists or writers is a better predictor than the year of creation or publication of their work. We anticipate that our dataset, especially combined with other information on artists, would be ideal to test whether such effects apply here too, at large scales.

## 7. Discussion and Conclusions

Massive, curated online datasets of artworks can be a powerful tool for art historians and cultural analysts. However, organised only by meta-data, it is difficult to perform image search, organisation and retrieval on aspects of images themselves, such as the color or style. In this paper, we describe and release the WikiArtVectors dataset, where we aim to bridge this gap. By releasing artworks with corresponding vectors, as well as aggregate vectors of artist, style, and genre, we provide a powerful framework for both retrieval/organisation, as well as cultural analysis. Our style and color representations are created using state-of-the-art methods in the fields of computer vision for retrieval purposes, and, when used in tandem with information-theoretic measures, can reveal novel patterns in cultural evolution. By providing this flexible but powerful temporal vector dataset of fine art, we contribute to the research efforts of art historians and digital humanists, as well as cultural analysts.

Future steps would be to add further vector representations and information to each artwork. For example, object-detection software can be used to tag artworks by content. With increasing variation in deep-neural-network-based image models, another addition would be to include style representations and image representations from various models. We note the limitations of vector representations in automatic discovery—as representations are often generated using specific machine learning tasks, they are not a substitute for close historical reading and analysis. Rather, these vectors and similar tools should be thought of as powerful tools to aid in discovery, and to create measurements to guide researchers to build theory (e.g., computational grounded theory [44]).

## Data Availability

We release our dataset and demo notebooks on GitHub: https://github.com/bhargavvader/WikiArtVectors, accessed on 9 June 2022.
